# Clinical trials for lupus nephritis. It is time for change

**DOI:** 10.1093/ckj/sfag016

**Published:** 2026-01-22

**Authors:** Enrique Morales, María Galindo

**Affiliations:** Department of Nephrology, Hospital Universitario 12 de Octubre; Research Institute Hospital 12 de Octubre (I+12); Complutense University of Madrid and 12 de Octubre University Hospital; RICORS2040-Renal, ISCIII, Madrid, Spain; Research Institute Hospital 12 de Octubre (I+12); Complutense University of Madrid and 12 de Octubre University Hospital; Department of Rheumatology, Hospital Universitario 12 de Octubre

To the Editor,

In the past 5 years, randomized trials with belimumab, voclosporin, and obinutuzumab have reshaped the therapeutic landscape of lupus nephritis (LN), improving complete renal response when added to background therapy. These data have been rapidly absorbed into contemporary guidelines (KDIGO 2024; ACR 2024/25), which now endorse early combination (‘triple’) immunosuppression in appropriate patients [1, 2].

However, the viability and external validity of LN clinical trials remain constrained by conventional eligibility criteria still built largely on ISN/RPS histologic class and proteinuria thresholds. BLISS-LN and AURORA-1 required class III/IV/V and predominantly proteinuria-driven composite endpoints; the phase 3 REGENCY programme followed similar contours. These axes, while practical, insufficiently capture the biological heterogeneity that underlies therapeutic response (Table 1) [3–5].

**Table 1:** Inclusion criteria for clinical trials.

Clinical trial	Renal histological class	Validation period for kidney biopsy	Proteinuria
Belimumab^3^	Active class III or IV (±V) or class V	≤6 months	UPCR ratio ≥1
Voclosporin^4^	Class III, IV, V (alone or in combination with class III or IV)	within 2 years of screening	UPCR of 1.5 mg/mg or more (or ≥2 mg/mg if pure class V)
Obinutuzumab^5^	Active class III or IV (±V)	≤6 months	UPCR ratio ≥1

UPCR, urinary protein creatinine ratio

First, class is different from activity. Two ‘class IV’ biopsies—one with an NIH activity index (AI) of 10 and chronicity index (CI) of 0, another with AI 2 and CI 4—are biologically and prognostically distinct. Multiple cohorts show AI/CI predict early response and long-term kidney outcomes better than class alone, arguing for AI/CI-anchored enrolment and stratification. Conversely, timing is critical when interpreting renal biopsy findings. A biopsy obtained within the preceding 4 weeks is likely to yield the most accurate and clinically relevant information.

Second, vascular pathology matters. Renal thrombotic microangiopathy is associated with higher acute dialysis needs and inferior renal survival in LN, yet vascular lesions are rarely inclusion or stratification variables. Vascular lesions are clearly associated with poorer outcomes and a greater likelihood of developing chronic kidney disease. Trials that ignore vascular involvement risk mixing pathophysiologic endotypes and diluting detectable benefit.

Third, proteinuria is necessary but not sufficient. A 12-month proteinuria target (<0.5 g/day) predicts long-term renal outcomes in LN, but proteinuria can be ‘immunologic’ (active inflammation/podocytopathy) or ‘non-immunologic’ (chronic scarring), with distinct therapeutic implications (escalating immunosuppression vs. nephro-/cardioprotection and SGLT2i, RAAS, BP, lipid targets). Accurate phenotyping of proteinuria in LN requires a clinicopathologic adjudication strategy, ideally complemented by urinary biomarker panels, to differentiate immunologic from non-immunologic mechanisms. Although proteinuria remains a key endpoint in clinical trials and routine practice, its interpretation must be contextualized. Current single biomarkers exhibit significant limitations; therefore, an integrated approach combining proteinuria and/or albuminuria, microscopic haematuria, and estimated GFR slope—alongside immunologic indicators such as anti-dsDNA titres and complement levels—provides a more robust framework. These parameters should be supplemented by histopathologic markers, including AI and CI, vascular lesions, and macrophage infiltration markers such as CD68, which may help elucidate the origin of proteinuria. Moreover, emerging urinary biomarkers strongly correlated with histologic findings—such as TGF-β1, PTX3, soluble CD163, CD11b, and IL-16—have demonstrated substantial predictive value, as highlighted in recent meta-analyses. Incorporating these multidimensional markers into phenotyping strategies may enhance risk stratification and optimize therapeutic decision making in LN [6].

A pragmatic blueprint to enhance the viability and personalization of forthcoming LN trials would include:

1. Eligibility anchored to AI/CI rather than class alone.
2. Mandatory capture of vascular lesions (thrombotic microangiopathy and other renal vascular lesions) for stratification and pre-specified subgroup analyses.
3. Phenotyping proteinuria mechanism (clinicopathologic adjudication ± urinary biomarker panels) to separate immunologic versus non-immunologic drivers; proteinuria remains a key endpoint, but context matters.
4. Phenotyping proteinuria mechanism (clinicopathologic adjudication ± urinary biomarker panels) to separate immunologic versus non-immunologic drivers; proteinuria remains a key endpoint, but context matters.
5. Establish re-biopsies at 12 months of immunosuppressive treatment to resolve doubts about clinical-histological dissociation.

Redesigning eligibility, stratification, and endpoints around activity, chronicity, vascular status, and proteinuria mechanisms will increase signal-to-noise, accelerate drug development, and align trial readouts with guideline implementation in real-world LN.
